# Supplementary material for: Radiomics for Discrimination between Early-Stage Nasopharyngeal Carcinoma and Benign Hyperplasia with Stable Feature Selection on MRI
Source: Cancers (Basel). 2022 Jul 14;14(14):3433. doi: 10.3390/cancers14143433 (PMC9324280; doi:10.3390/cancers14143433)
Supplement: Supplementary file 1 [file cancers-14-03433-s001.zip › Supplementary Text, Figures and Tables.docx]

Supplementary Materials: Radiomics for Discrimination between Early-Stage Nasopharyngeal Carcinoma and Benign Hyperplasia with Stable Feature Selection on MRI

Lun M. Wong ^1^, Qi Yong H. Ai ^1,2,^*, Rongli Zhang ^1^, Frankie Mo ^3^ and Ann D. King ^1,^*

Text S1. PyRadiomics setting

PyRadiomics v3.0.1 [1] was used for radiomic feature extraction. The setting file used is as follows:

imageType:

Original: {}

LBP2D:

lbp2DRadius: 0.9 # eqauls to 2 x pixel size

lbp2DMethod: 'default'

force2D: True

LBP3D:

lbp3DIcosphereRadius : 0.9 # eqauls to 2 x pixel size

LoG:

sigma: [0.4492]

Gradient: {}

Exponential: {}

featureClass:

firstorder:

- Energy

- Entropy

- Kurtosis

- Maximum

- MeanAbsoluteDeviation

- Mean

- RobustMeanAbsoluteDeviation

- RootMeanSquared

- Skewness

- Uniformity

- Variance

shape:

glcm:

glrlm:

setting:

resampledPixelSpacing: [0.4492, 0.4492, 0.4492]

binWidth: 1

The resampled pixel spacing was decided according to the mode of pixel sizes of all involved MRI scans (0.4492 × 0.4492 mm), and the slice thickness was resampled such that the voxels of the image volume were isometric. The complete list of 422 extracted features is provided in the sheet “List of features” in the Excel file: Datasheet S1.xlsx.

Text S2. Details of the Bagged-Boosted RENT (BB-RENT):

BB-RENT is based on the repeated elastic net technique (RENT) proposed by Jenul et al. [2]. The pseudo-code of the original RENT is provided here for reference.

**Algorithm S1.** Repeated elastic net technique (RENT).

*Declare:*

$X\in\mathbb{R}^{W\times M}$, matrix of *M* input features of *W* patients

$Y\in\mathbb{R}^{W}$, vector of class label of the *W* patients, each element is either 0 or 1

$K\in\mathbb{Z}^{+}$, number of iterations of elastic nets trained

$\gamma\in[0, 1]$, resampling percentage

$B\in\mathbb{R}^{K\times M}$, matrix of Enet derived coefficients

$\beta_{k}\in\mathbb{R}^{M}$, the row vector of coefficients of the *k*^th^ model

$\beta_{(m)} \in\mathbb{R}^{K},$ the column vector of coefficient of the *m*^th^ covariate

*Algorithm:*

**for** $k$ in $\left[ 1,K \right]$

$\left\{ X^{'}, Y^{'} \right\}=Resample(X, Y;\gamma)$ *# randomly sample* $\gamma\%$ *of all patients*

$\beta_{k}=ENET(X', Y')$ *# obtain covariates coefficients by Enet*

Put $\beta_{k}$ into the *k*^th^ row of $B$

**endfor**

returnedList = [ ]

**for** $m$ in $[1, M]$ *# iterate each unique feature*

$\tau_{1(m)}=$ $M^{-1}\sum_{m=1}^{M} one[\beta_{\left( m \right)}\neq0]$ *# P(coef.* $\neq$ *0)*

$\tau_{2(m)}=M^{-1}\left| \sum_{m=1}^{M} \mathrm{sign}\left[ \beta_{\left( m \right)} \right] \right|$ *# P(coef. have stable sign)*

$\tau_{3(m)}=ttest(\beta_{\left( m \right)}, 0)$ # *T-test for H_0_: coef. mean = 0*

**if** all $\tau_{i(m)}$ > threshold_i_:

returnedList.append(*m*^th^ feature)

**endif**

**endfor**

*Return:*

returnedList

In this study, we propose to improve RENT through a combination of bagging and boosting. Bagging (or bootstrap aggregation) generally refers to aggregating a set of “learners” that are trained using different portions of data bootstrapped from an original data pool. Bagging has been shown to reduce the variance in model performance and improve its robustness. Bootstrapping is also frequently employed to estimate the statistical characteristics of samples, such as 95% confidence intervals. Boosting refers to the aggregation of weak learners that are usually clones of the same classifier trained in sequence on the same batch of data. Each weak learner in a boosting chain learns from the mistakes made by its predecessors by adjusting the sample weights prior to its training. The chain generally stops when its length reaches an arbitrary maximum or when the loss converges. The proposed bagged-boosted RENT (BB-RENT) first boosts and then bags the original RENT. The pseudo-code of the proposed BB-RENT is as follows:

**Algorithm S2.** Bagged-Boosted RENT (BB-RENT).

*Declare:*

${Q\mathbb{\in Z}}^{+}$, number of times the data are bootstrapped

${N\mathbb{\in Z}}^{+}$, maximum depth of boosting the elastic net

$\alpha\in\mathbb{R}^{N}$, boosting coefficients vector with between 1 to *N* elements

$\beta_{k}^{'}\boldsymbol{\in}\mathbb{R}^{M\times N}$, coefficients of boosted elastic nets, between 1 to *N* columns

each column is a set of coefficients for the covariates

$\eta\in[0, 1]$, threshold percentage criterion for including feature in to selected list

*[For the rest, please reference Algorithm 1]*

*Algorithm:*

outerList = [ ]

**for** $q$ in $\left[ 1,Q \right]$

**for** $k$ in $\left[ 1,K \right]$

$\left\{ X^{'}, Y^{'} \right\}=Resample(X, Y;\gamma)$

$\alpha,\beta_{k}^{'}=Boosted[ENET\left( X^{'}, Y^{'} \right)]$ *# max. boost depth is N*

$\beta_{k}=sum\_column(\alpha{\beta^{'}}_{k}^{T})$ *# sum weighted by boost coefficients*

Put $\beta_{k}$ into the *k*^th^ row of $B$

**endfor**

innerList = [ ]

**for** $m$ in $[1, M]$

Evaluate $\tau_{1}$, $\tau_{2}$ and $\tau_{3}$

**if** all $\tau_{i(m)}$ > threshold_i_:

innerList.append(*m*^th^ feature)

**endif**

**endfor**

outerList.append(innerList)

**end**

returnedList = []

**for** $m$ in $[1, M]$

count freqeuncies of *m*^th^ feature in outerList

**if** frequency > $\eta\cdot Q$

returnedList.append(*m*^th^ feature)

**endif**

**endfor**

*Return:*

returnedList

To facilitate understanding, the formulation of the boosted elastic net is presented as follows:

| $y=\sum_{n=1}^{N} \alpha_{n}X\beta_{n},$ |  |
| --- | --- |

where $y$ is the prediction, $X$ is the input feature matrix, $\alpha_{n}$ is the boosting coefficients obtained using Adaboost [3], $\beta_{n}$ is the coefficients for each feature fitted using elastic net, the subscript $n$ is the index for each weak learner (an elastic net) in the boost chain, and $N$ is the maximum depth for Adaboost. The original RENT is equivalent to setting $N=1$, and the fitting of $\beta_{1}$ was repeated $K$ times with different subsets of the data (i.e., $X$ varied) in order to estimate the statistical properties of $\beta_{1}$. In this study, we set $N = 25$ and summed the coefficients of each weaker learner weighted by the corresponding boosting coefficient, $\beta^{'}=\alpha_{1}\beta_{1}+\alpha_{2}\beta_{2}+\ldots+\alpha_{N}\beta_{N}$. The statistical properties of $\beta'$ were accessed through $\tau_{1}$, $\tau_{2}$, and $\tau_{3}$ in each bootstrapped iteration of BB-RENT, as opposed to just $\beta_{1}$ in the original RENT.

We further bagged the boosted RENT by bootstrapping $Q$ times and aggregating the $Q$ sets of features to ultimately nominate those that were selected for more than $\eta\cdot Q$ times, with $\eta=50\%$ and $Q=150$.

Text S3. List of Hyperparameters and Their Values for Model Training.

As covered in the main text, there were two major parts in this study involving hyperparameter tuning: the feature selection step and the model-building step.

For feature selection, the proposed BB-RENT was based on elastic net. Therefore, an extensive grid search was conducted to identify the best hyperparameter for elastic net based on maximization of the classification performance, as per the suggestion of Jenul et al. [2]. The available hyperparameters for elastic net were the L1 ratio and the regularization factor, $\alpha$. A grid search of these two parameters was performed using the Scikit-learn v1.0.1 package [4], in which the GridSearchCV function was invoked. This function allows users to perform a customized grid search to identify the best-performing set of hyperparameters for each classification model. The grid search identified L1-ratio = 0.5 and $\alpha$ = 0.02 (Figure S1) as having one of the highest classification performances, where the curve of $\alpha$ = 0.02 showed better stability than the rest of the combination (i.e., a less wavy curve) and also led to a high performance score. This combination was fixed and used in both BB-RENT and the original RENT in our experiments for feature selection.

The structured dictionary used for the grid search in the model-building step is provided below:

clf = pipeline.*Pipeline***(**[
 **(**'standardization', preprocessing.*StandardScaler***())**,
 **(**'classification', 'passthrough'**)**]**)**# Construct tests to perform
param_grid_dict = {
 'Support Vector Regression': {
 'classification': [svm.*SVR***(**tol=1E-4, max_iter=-1**)**],
 'classification__kernel': ['linear', 'poly', 'rbf', 'sigmoid'],
 'classification__C': [1, 10, 100, 1000],
 'classification__degree': [3, 5, 7, 9],
 'classification__epsilon': [1, 0.1, 0.01]
 },
 'Logistic Regression': {
 'classification': [linear_model.*LogisticRegression***(**penalty='elasticnet',
 solver='saga', tol=1E-5,
 max_iter=5500,
 verbose=verbose**)**],
 'classification__C': [0.1, 1, 10, 100, 1000],
 'classification__l1_ratio': [0.1, 0.3, 0.5, 0.7, 0.9]
 },
 'Random Forest': {
 'classification': [ensemble.*RandomForestRegressor***(**n_estimators=50**)**],
 'classification__criterion': ['squared_error', 'poisson']
 },
 'Perceptron': {
 'classification': [neural_network.*MLPRegressor***(**learning_rate='adaptive',
 tol=1E-4,
 max_iter=5000,
 verbose=verbose**)**],
 'classification__hidden_layer_sizes': [**(**100**)**, **(**20, 50, 100**)**, **(**100, 50, 20**)**],
 'classification__learning_rate_init': [1, 0.1, 1E-2, 1E-3, 1E-4]
 },
 'KNN': {
 'classification': [neighbors.*KNeighborsRegressor***(**n_jobs=5**)**],
 'classification__n_neighbors': [3, 5, 10, 20],
 }
}

The best parameters identified by the GridSearchCV function for each method were tabulated. Each fold showed slight variations, but support vector regression (SVR) and random forest (RF) remained quite stable, whereas logistic regression showed the greatest variations (Table S1).


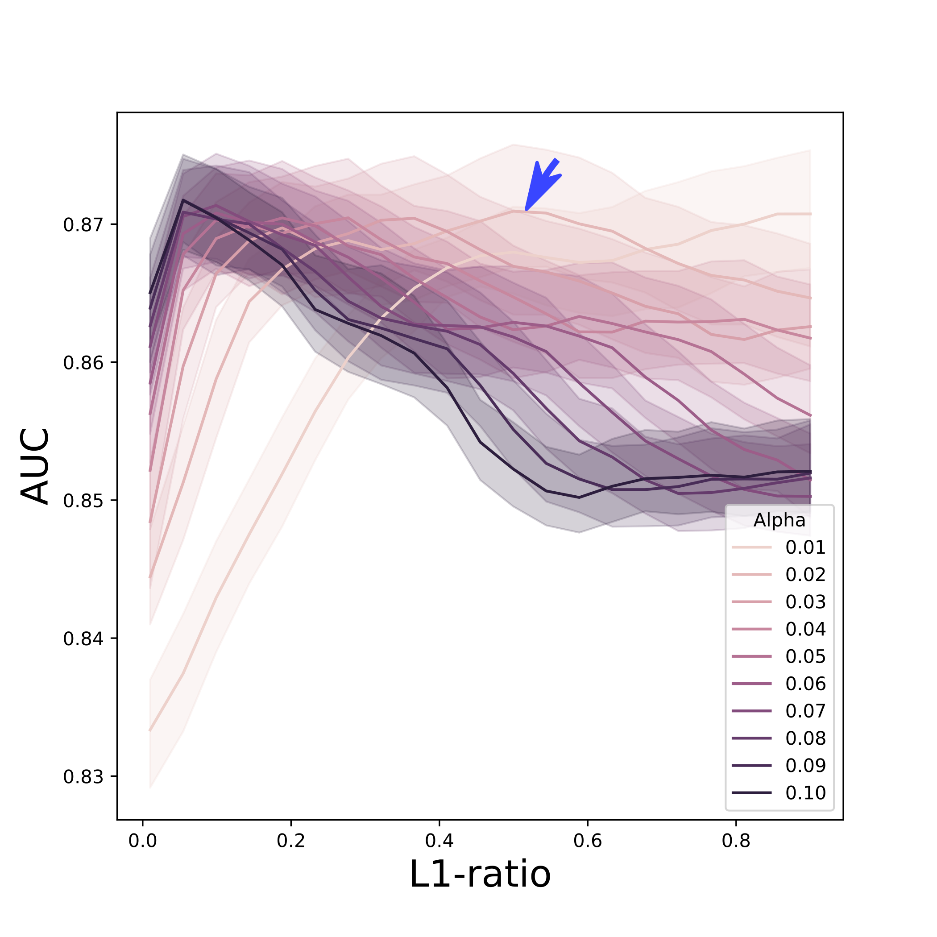


**Figure S1.** Grid search performed with all data from the 3T cohort for the optimal hyperparameters of feature extraction using elastic net. The grid search was repeated 10 times to establish the error margin (transparent bands). The grid search identified L1 ratio = 0.5 and α = 0.02 (blue arrow) as presenting the best classification performance based on the area under the curve (AUC). Some other combinations also demonstrated high AUC values, but their curves appeared to be less stable than that with α = 0.02.

**Table S1.** Best hyperparameters obtained from the grid search for model building. Support vector regression not only showed the best performance but also demonstrated better stability than other models during hyperparameter tuning. C is the regularization parameter; epsilon for support vector regression (SVR) is the soft-margin tolerance; the degree of SVR is greyed, as this hyperparameter was actually omitted by linear SVR; and α of the elastic net is the regularization parameter. RF = random forest, kNN = k-nearest neighbors.

| Model | Hyperparameters | Fold 1 | Fold 2 | Fold 3 | Fold 4 | Fold 5 |
| --- | --- | --- | --- | --- | --- | --- |
| SVR | C | 1 | 1 | 1 | 1 | 1 |
|  | degree | 3 | 3 | 3 | 3 | 3 |
|  | epsilon | 0.1 | 0.01 | 0.01 | 0.1 | 0.01 |
|  | kernel | linear | linear | linear | linear | linear |
| Logistic Regression | C | 1.0 | 100.0 | 1.0 | 100.0 | 10.0 |
|  | l1_ratio | 0.1 | 0.1 | 0.3 | 0.1 | 0.1 |
| RF | criterion | squared_error | squared_error | squared_error | squared_error | squared_error |
| Perceptron | hidden_layer_sizes | (100, 50, 20) | (100, 50, 20) | (100, 50, 20) | (20, 50, 100) | (100, 50, 20) |
|  | learning_rate_init | 0.0001 | 0.0001 | 0.0001 | 0.0001 | 0.0001 |
| KNN | n_neighbors | 20 | 20 | 20 | 20 | 20 |

Text S2. Metrics for Measuring Stability

The Jaccard index (JAC) was used to measure the stability of a feature selection method in this study. As the JAC measures the similarity between two sets of features only, the arithmetic mean of the pairwise JAC, $\bar{\mathrm{JAC}}$, was evaluated and analysed, defined as:

| $\mathrm{JAC}\left( X,Y \right)=\frac{\left\vert X\cup Y \right\vert}{\left\vert X\cap Y \right\vert},$ |  |
| --- | --- |
| $\bar{\mathrm{JAC}}\left( \mathbf{X} \right)=\left[ \frac{B!}{\left( B-2 \right)!} \right]^{-1}\sum_{\begin{aligned} X_{i},X_{j}\in\mathbf{X} \\ i\neq j \end{aligned}} \mathrm{JAC}(X_{i}, X_{j}),$ |  |

where $\mathbf{X=}\left\{ X_{1},X_{2},\ldots X_{B} \right\}$ is the collection of feature subset $X_{i}$ nominated in the *i*^th^ run of the stability test. In a sense, this arithmetic mean of the JAC address the average pairwise similarity rather than the similarity among many feature subsets; thus, it might not accurately reflect the stability of the feature selection methods.

Considering this weakness of the JAC, we also explored alternative measures that can address the stability by quantifying the similarity of a set with more than two sets of features. Nogueira’s score (NS) was proposed to address this issue, defined as [5]:

| $NS\left( \mathcal{Z} \right)=1-\frac{\frac{1}{d}\sum_{f=1}^{d} s_{f}^{2}}{\frac{\bar{k}}{d}\left( 1-\frac{\bar{k}}{d} \right)},$ |  |
| --- | --- |

where $\mathcal{Z}$ is a $B\times d$ Boolean matrix with $B=100$ sets of features selected from a pool of $d$ features, with 1 indicating that the feature was selected and 0 otherwise; $\bar{k}$ is the mean number of features selected in each of the $M$ runs; $s_{f}=\frac{M}{M-1}\hat{p}_{f}(1-\hat{p}_{f})$ is the unbiased sample variance of the selection probability of selecting the $f$^th^ feature; and $\hat{p}_{f}$ is the observed selection probability of the $f$^th^ feature. The value of NS ranges from 0 to 1, with a higher value indicating a better match among the involved sets.

Text S4. Detailed Results of Feature Selection Stability Evaluation

Supervised feature selection techniques, including (i) a single run of elastic net, (ii) the repeated elastic net technique (RENT), (iii) boosted RENT, (iv) bagged RENT, and (v) the proposed bagged-boosted RENT (BB-RENT), were repeated 100 times to select radiomic features extracted from 80% of patients randomly sampled from the 3T cohort. The selected features in each run were analysed and summarized based on their frequency of selection; this frequency also empirically represents the probability of the feature being selected, such that a higher frequency suggests higher relevance and stability. A summary of the frequency of each feature that was ranked in the top 15 among the 100 feature selection runs for the 5 different methods is plotted in Figure S2, and a summary of the features selected across the 100 runs for each method is tabulated in Table S2. The full list of radiomic features selected in each run by each method is provided in the corresponding datasheets in the Excel file: Datasheet S1.xlsx.


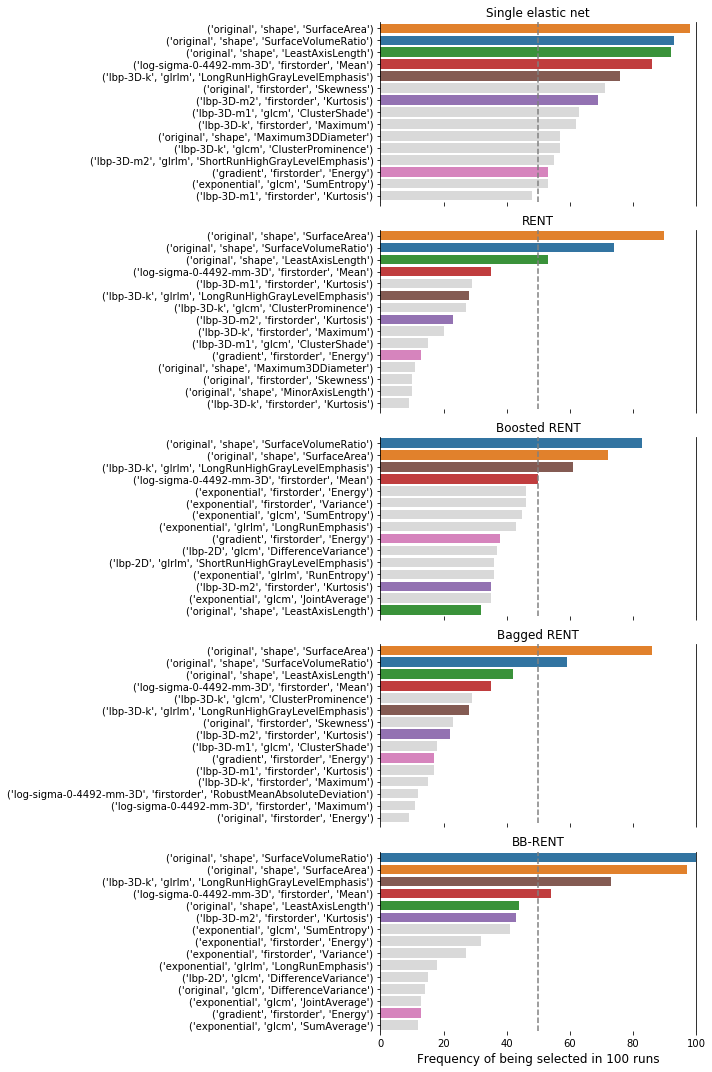


**Figure S2.** Selection frequencies of various features by the five methods across 100 repeated runs, each graph showing only the top 15 features with the highest frequencies out of the total of 422 features. The intersection of these five methods gives seven features, which are color-coded. The dotted line indicates the mid-point of the graphs. The feature names are presented in the format ([imaging filter], [feature category], [feature name]), following the PyRadiomics convention. Compared to the original RENT, the proposed BB-RENT increased the selection frequency of top-ranked features while maintaining a lower frequency for lower-ranked features, ultimately contributing to an improvement in feature selection stability. RENT = repeated elastic net technique, BB-RENT = bagged-boosted RENT, lbp = local binary pattern, log = Laplacian of Gaussian, 3D = three-dimensional.

**Table S2.** Summary of the selection frequencies of radiomic features by each method over 100 resampled runs. Of the 3T cohort, 80% was resampled at random in each of the 100 runs in order to introduce data variations such that the feature selection stability of the five methods could be tested. Only features that were selected at least once by any of the five methods are included in this table. RENT = repeated elastic net technique, BB-RENT = bagged-boosted RENT, glcm = grey-level co-occurrence matrix, glrlm = grey-level run-length matrix, lbp = local binary pattern, log = Laplacian of Gaussian.

| Filter Type | Feature Type | Feature Name | Single Elastic Net | RENT | Boosted RENT | Bagged RENT | BB-RENT |
| --- | --- | --- | --- | --- | --- | --- | --- |
| exponential | firstorder | Energy | 32 | 6 | 46 | 6 | 32 |
|  |  | Entropy | 5 | 0 | 6 | 0 | 0 |
|  |  | Kurtosis | 2 | 0 | 1 | 0 | 0 |
|  |  | Maximum | 8 | 0 | 6 | 1 | 1 |
|  |  | Mean | 2 | 0 | 2 | 0 | 0 |
|  |  | MeanAbsoluteDeviation | 1 | 0 | 11 | 0 | 0 |
|  |  | RobustMeanAbsoluteDeviation | 5 | 0 | 14 | 0 | 0 |
|  |  | RootMeanSquared | 0 | 0 | 2 | 0 | 0 |
|  |  | Skewness | 4 | 1 | 4 | 0 | 0 |
|  |  | Variance | 27 | 1 | 46 | 2 | 27 |
|  | glcm | Autocorrelation | 4 | 0 | 7 | 0 | 0 |
|  |  | ClusterProminence | 8 | 0 | 14 | 0 | 0 |
|  |  | ClusterShade | 33 | 6 | 8 | 1 | 0 |
|  |  | ClusterTendency | 27 | 2 | 29 | 4 | 1 |
|  |  | Contrast | 8 | 0 | 8 | 0 | 0 |
|  |  | DifferenceAverage | 3 | 0 | 8 | 0 | 0 |
|  |  | DifferenceEntropy | 5 | 0 | 10 | 0 | 0 |
|  |  | DifferenceVariance | 4 | 0 | 10 | 1 | 0 |
|  |  | JointAverage | 28 | 3 | 35 | 0 | 13 |
|  |  | JointEntropy | 20 | 0 | 18 | 2 | 2 |
|  |  | SumAverage | 28 | 3 | 32 | 0 | 12 |
|  |  | SumEntropy | 53 | 5 | 45 | 1 | 41 |
|  |  | SumSquares | 13 | 0 | 17 | 0 | 0 |
|  | glrlm | GrayLevelNonUniformity | 8 | 0 | 16 | 0 | 0 |
|  |  | GrayLevelVariance | 41 | 6 | 20 | 0 | 3 |
|  |  | HighGrayLevelRunEmphasis | 1 | 0 | 16 | 0 | 0 |
|  |  | LongRunEmphasis | 27 | 4 | 43 | 0 | 18 |
|  |  | LongRunHighGrayLevelEmphasis | 11 | 0 | 9 | 0 | 0 |
|  |  | LongRunLowGrayLevelEmphasis | 5 | 0 | 3 | 0 | 0 |
|  |  | RunEntropy | 31 | 4 | 36 | 8 | 2 |
|  |  | RunLengthNonUniformity | 5 | 0 | 12 | 0 | 2 |
|  |  | RunVariance | 22 | 5 | 19 | 1 | 6 |
|  |  | ShortRunHighGrayLevelEmphasis | 17 | 0 | 25 | 0 | 1 |
| gradient | firstorder | Energy | 53 | 13 | 38 | 17 | 13 |
|  |  | Entropy | 1 | 0 | 7 | 0 | 0 |
|  |  | Maximum | 0 | 0 | 1 | 0 | 0 |
|  |  | MeanAbsoluteDeviation | 3 | 0 | 0 | 0 | 0 |
|  |  | RobustMeanAbsoluteDeviation | 3 | 0 | 21 | 0 | 0 |
|  |  | Variance | 2 | 0 | 6 | 0 | 0 |
|  | glcm | Autocorrelation | 0 | 0 | 4 | 0 | 0 |
|  |  | ClusterProminence | 8 | 0 | 8 | 0 | 0 |
|  |  | ClusterShade | 4 | 0 | 3 | 0 | 0 |
|  |  | ClusterTendency | 10 | 1 | 11 | 1 | 1 |
|  |  | Contrast | 2 | 0 | 6 | 0 | 0 |
|  |  | DifferenceAverage | 1 | 0 | 1 | 0 | 0 |
|  |  | DifferenceEntropy | 0 | 0 | 3 | 0 | 0 |
|  |  | DifferenceVariance | 7 | 0 | 11 | 0 | 0 |
|  |  | JointAverage | 0 | 0 | 3 | 0 | 0 |
|  |  | JointEntropy | 0 | 1 | 2 | 0 | 0 |
|  |  | SumAverage | 0 | 0 | 4 | 0 | 0 |
|  |  | SumEntropy | 1 | 0 | 6 | 0 | 0 |
|  |  | SumSquares | 1 | 0 | 3 | 0 | 0 |
|  | glrlm | GrayLevelNonUniformity | 16 | 0 | 2 | 0 | 0 |
|  |  | GrayLevelVariance | 3 | 0 | 7 | 0 | 0 |
|  |  | HighGrayLevelRunEmphasis | 0 | 0 | 7 | 0 | 0 |
|  |  | LongRunHighGrayLevelEmphasis | 0 | 0 | 7 | 0 | 0 |
|  |  | RunEntropy | 3 | 0 | 10 | 0 | 0 |
|  |  | RunLengthNonUniformity | 2 | 0 | 0 | 0 | 0 |
|  |  | ShortRunHighGrayLevelEmphasis | 0 | 0 | 6 | 0 | 0 |
| lbp-2D | firstorder | Energy | 9 | 0 | 6 | 0 | 0 |
|  |  | Kurtosis | 1 | 0 | 0 | 0 | 0 |
|  |  | Mean | 2 | 0 | 5 | 0 | 0 |
|  |  | MeanAbsoluteDeviation | 17 | 1 | 24 | 2 | 1 |
|  |  | RobustMeanAbsoluteDeviation | 23 | 3 | 11 | 2 | 0 |
|  |  | Skewness | 3 | 0 | 3 | 0 | 0 |
|  |  | Variance | 10 | 1 | 8 | 0 | 0 |
|  | glcm | Autocorrelation | 34 | 3 | 24 | 0 | 1 |
|  |  | ClusterProminence | 8 | 0 | 1 | 0 | 0 |
|  |  | ClusterShade | 23 | 0 | 15 | 1 | 0 |
|  |  | ClusterTendency | 22 | 0 | 19 | 0 | 1 |
|  |  | Contrast | 5 | 0 | 21 | 0 | 0 |
|  |  | DifferenceAverage | 20 | 2 | 20 | 0 | 0 |
|  |  | DifferenceVariance | 25 | 1 | 37 | 0 | 15 |
|  |  | JointAverage | 17 | 0 | 11 | 0 | 0 |
|  |  | JointEntropy | 16 | 3 | 12 | 0 | 0 |
|  |  | SumAverage | 17 | 0 | 13 | 0 | 0 |
|  |  | SumSquares | 15 | 1 | 15 | 1 | 0 |
|  | glrlm | GrayLevelVariance | 7 | 0 | 5 | 0 | 0 |
|  |  | HighGrayLevelRunEmphasis | 1 | 0 | 10 | 0 | 0 |
|  |  | LongRunHighGrayLevelEmphasis | 16 | 0 | 13 | 0 | 0 |
|  |  | RunLengthNonUniformity | 4 | 0 | 0 | 0 | 0 |
|  |  | ShortRunHighGrayLevelEmphasis | 7 | 0 | 36 | 0 | 5 |
| lbp-3D-k | firstorder | Energy | 13 | 0 | 2 | 0 | 0 |
|  |  | Kurtosis | 42 | 9 | 14 | 4 | 0 |
|  |  | Maximum | 62 | 20 | 16 | 15 | 0 |
|  | glcm | ClusterProminence | 57 | 27 | 23 | 29 | 0 |
|  | glrlm | GrayLevelNonUniformity | 1 | 0 | 0 | 0 | 0 |
|  |  | LongRunEmphasis | 6 | 0 | 12 | 0 | 0 |
|  |  | LongRunHighGrayLevelEmphasis | 76 | 28 | 61 | 28 | 73 |
|  |  | LongRunLowGrayLevelEmphasis | 15 | 0 | 22 | 0 | 0 |
|  |  | RunLengthNonUniformity | 1 | 0 | 3 | 0 | 0 |
|  |  | RunVariance | 16 | 1 | 12 | 1 | 0 |
| lbp-3D-m1 | firstorder | Energy | 22 | 0 | 0 | 0 | 0 |
|  |  | Kurtosis | 48 | 29 | 23 | 17 | 8 |
|  |  | Variance | 0 | 0 | 1 | 0 | 0 |
|  | glcm | Autocorrelation | 6 | 0 | 17 | 0 | 0 |
|  |  | ClusterProminence | 14 | 1 | 22 | 0 | 5 |
|  |  | ClusterShade | 63 | 15 | 22 | 18 | 1 |
|  |  | ClusterTendency | 6 | 0 | 4 | 0 | 0 |
|  |  | Contrast | 14 | 0 | 14 | 0 | 0 |
|  |  | DifferenceAverage | 3 | 0 | 1 | 0 | 0 |
|  |  | DifferenceVariance | 21 | 0 | 23 | 0 | 0 |
|  |  | SumAverage | 13 | 1 | 22 | 1 | 0 |
|  |  | SumSquares | 4 | 0 | 1 | 0 | 0 |
|  | glrlm | GrayLevelNonUniformity | 0 | 0 | 1 | 0 | 0 |
|  |  | GrayLevelVariance | 4 | 0 | 2 | 0 | 0 |
|  |  | HighGrayLevelRunEmphasis | 19 | 2 | 5 | 1 | 0 |
|  |  | RunLengthNonUniformity | 1 | 0 | 0 | 0 | 0 |
|  |  | ShortRunHighGrayLevelEmphasis | 8 | 0 | 16 | 0 | 0 |
| lbp-3D-m2 | firstorder | Energy | 12 | 0 | 3 | 0 | 0 |
|  |  | Kurtosis | 69 | 23 | 35 | 22 | 43 |
|  |  | Maximum | 25 | 3 | 5 | 3 | 0 |
|  |  | Mean | 14 | 0 | 6 | 0 | 0 |
|  |  | RootMeanSquared | 10 | 0 | 0 | 0 | 0 |
|  | glcm | Autocorrelation | 1 | 0 | 9 | 0 | 0 |
|  |  | ClusterProminence | 8 | 0 | 20 | 0 | 3 |
|  |  | ClusterShade | 46 | 7 | 10 | 6 | 0 |
|  |  | ClusterTendency | 7 | 0 | 12 | 0 | 0 |
|  |  | Contrast | 6 | 0 | 15 | 0 | 1 |
|  |  | DifferenceAverage | 1 | 0 | 2 | 0 | 0 |
|  |  | DifferenceVariance | 17 | 2 | 17 | 0 | 1 |
|  |  | JointAverage | 4 | 0 | 18 | 0 | 0 |
|  |  | SumAverage | 4 | 0 | 21 | 0 | 0 |
|  |  | SumSquares | 2 | 0 | 2 | 0 | 0 |
|  | glrlm | GrayLevelVariance | 18 | 1 | 17 | 0 | 0 |
|  |  | HighGrayLevelRunEmphasis | 37 | 2 | 7 | 0 | 0 |
|  |  | LongRunHighGrayLevelEmphasis | 2 | 0 | 1 | 0 | 0 |
|  |  | ShortRunHighGrayLevelEmphasis | 55 | 4 | 18 | 2 | 3 |
| log-sigma-0-4492-mm-3D | firstorder | Energy | 2 | 0 | 19 | 0 | 0 |
|  |  | Entropy | 10 | 0 | 6 | 0 | 0 |
|  |  | Kurtosis | 16 | 0 | 11 | 0 | 0 |
|  |  | Maximum | 16 | 8 | 6 | 11 | 0 |
|  |  | Mean | 86 | 35 | 50 | 35 | 54 |
|  |  | MeanAbsoluteDeviation | 4 | 0 | 0 | 0 | 0 |
|  |  | RobustMeanAbsoluteDeviation | 48 | 7 | 26 | 12 | 3 |
|  |  | RootMeanSquared | 5 | 0 | 5 | 0 | 0 |
|  |  | Skewness | 15 | 0 | 10 | 2 | 0 |
|  |  | Variance | 3 | 0 | 7 | 0 | 0 |
|  | glcm | Autocorrelation | 5 | 0 | 8 | 1 | 0 |
|  |  | ClusterProminence | 29 | 1 | 15 | 0 | 0 |
|  |  | ClusterShade | 17 | 0 | 5 | 0 | 0 |
|  |  | ClusterTendency | 1 | 0 | 10 | 0 | 0 |
|  |  | Contrast | 4 | 0 | 9 | 0 | 1 |
|  |  | DifferenceAverage | 5 | 0 | 12 | 0 | 0 |
|  |  | DifferenceEntropy | 4 | 0 | 12 | 0 | 0 |
|  |  | DifferenceVariance | 3 | 0 | 10 | 0 | 0 |
|  |  | JointAverage | 22 | 0 | 15 | 1 | 0 |
|  |  | JointEntropy | 5 | 0 | 0 | 0 | 0 |
|  |  | SumAverage | 22 | 0 | 13 | 1 | 0 |
|  |  | SumEntropy | 12 | 0 | 5 | 2 | 0 |
|  |  | SumSquares | 1 | 0 | 4 | 0 | 0 |
|  | glrlm | GrayLevelNonUniformity | 9 | 0 | 3 | 0 | 0 |
|  |  | GrayLevelVariance | 3 | 0 | 5 | 0 | 0 |
|  |  | HighGrayLevelRunEmphasis | 4 | 0 | 6 | 0 | 0 |
|  |  | LongRunEmphasis | 23 | 0 | 8 | 0 | 0 |
|  |  | LongRunHighGrayLevelEmphasis | 12 | 0 | 10 | 0 | 0 |
|  |  | RunLengthNonUniformity | 3 | 0 | 3 | 0 | 0 |
|  |  | RunVariance | 32 | 1 | 6 | 0 | 0 |
|  |  | ShortRunHighGrayLevelEmphasis | 9 | 0 | 8 | 0 | 1 |
| original | firstorder | Energy | 35 | 0 | 14 | 9 | 0 |
|  |  | Entropy | 0 | 0 | 1 | 0 | 0 |
|  |  | Kurtosis | 38 | 2 | 16 | 3 | 0 |
|  |  | Maximum | 13 | 1 | 19 | 0 | 0 |
|  |  | Mean | 38 | 3 | 14 | 2 | 0 |
|  |  | MeanAbsoluteDeviation | 0 | 0 | 1 | 0 | 0 |
|  |  | RobustMeanAbsoluteDeviation | 21 | 1 | 15 | 1 | 0 |
|  |  | RootMeanSquared | 24 | 0 | 18 | 0 | 0 |
|  |  | Skewness | 71 | 10 | 17 | 23 | 0 |
|  |  | Variance | 3 | 0 | 10 | 0 | 0 |
|  | glcm | Autocorrelation | 0 | 0 | 1 | 0 | 0 |
|  |  | ClusterProminence | 19 | 0 | 2 | 0 | 0 |
|  |  | ClusterShade | 26 | 5 | 16 | 6 | 0 |
|  |  | ClusterTendency | 14 | 1 | 16 | 0 | 0 |
|  |  | Contrast | 13 | 0 | 14 | 0 | 0 |
|  |  | DifferenceAverage | 1 | 0 | 3 | 0 | 0 |
|  |  | DifferenceEntropy | 1 | 0 | 3 | 0 | 0 |
|  |  | DifferenceVariance | 34 | 1 | 29 | 3 | 14 |
|  |  | JointEntropy | 1 | 0 | 4 | 0 | 0 |
|  |  | SumEntropy | 3 | 0 | 12 | 0 | 0 |
|  |  | SumSquares | 14 | 1 | 17 | 0 | 0 |
|  | glrlm | GrayLevelNonUniformity | 8 | 0 | 3 | 0 | 0 |
|  |  | GrayLevelVariance | 9 | 0 | 10 | 0 | 0 |
|  |  | HighGrayLevelRunEmphasis | 0 | 0 | 1 | 0 | 0 |
|  |  | LongRunHighGrayLevelEmphasis | 0 | 0 | 1 | 0 | 0 |
|  |  | RunEntropy | 22 | 2 | 17 | 1 | 1 |
|  |  | RunLengthNonUniformity | 6 | 0 | 1 | 0 | 0 |
|  | shape | LeastAxisLength | 92 | 53 | 32 | 42 | 44 |
|  |  | MajorAxisLength | 41 | 3 | 21 | 4 | 3 |
|  |  | Maximum2DDiameterColumn | 19 | 0 | 14 | 0 | 0 |
|  |  | Maximum2DDiameterRow | 10 | 2 | 10 | 4 | 0 |
|  |  | Maximum2DDiameterSlice | 17 | 1 | 22 | 3 | 0 |
|  |  | Maximum3DDiameter | 57 | 11 | 24 | 3 | 3 |
|  |  | MinorAxisLength | 1 | 10 | 4 | 5 | 0 |
|  |  | SurfaceArea | 98 | 90 | 72 | 86 | 97 |
|  |  | SurfaceVolumeRatio | 93 | 74 | 83 | 59 | 100 |

Text S5. Standardization Parameters

Different radiomic features naturally have different population means and variances, which may pose challenges during model fitting. For example, if the features involved present very different means and variances, those with a large mean could easily dominate other features and make the model biased. Therefore, it is a necessary practice to standardize the features based on their mean and variance. This was achieved using the method preprocessing. StandardScaler of the scikit-learn package [4], which standardizes the features by subtraction of sample mean, $\mu$, and scaling based on sample variance, $\sigma$:

| $X^{'}=\frac{X-\mu}{\sigma}.$ |  |
| --- | --- |

The mean ($\mu$) and variance $(\sigma$) (scale) of each feature were computed from the training data during model fitting and subsequently used to standardize the validation data. The means and variances fitted using the 3T MRI training cohort during fivefold cross validation are tabulated below (Table S3).

**Table S3.** Mean and variance used for standardization of radiomic features in each fold. Sample variances were normalized to 1 using the scaling factors shown.

| Filter Type | Feature Type | | Feature Name | | Fold 1 | | Fold 2 | | | | Fold 3 | | |
| --- | --- | --- | --- | --- | --- | --- | --- | --- | --- | --- | --- | --- | --- |
|  |  |  |  |  | **mean** | **scale** | **mean** | | **scale** | | **mean** | | **scale** |
| exponential | firstorder | | Energy | | - | - | 3.11 × 10^6^ | | 2.80 × 10^6^ | | - | | - |
|  |  |  | Variance | | - | - | - | | - | | - | | - |
|  | glcm | | SumEntropy | | 4.61 × 10^0^ | 5.99 × 10^−1^ | - | | - | | - | | - |
|  | glrlm | | RunVariance | | - | - | 1.32 × 10^0^ | | 1.12 × 10^0^ | | - | | - |
| gradient | firstorder | | Energy | | 6.80 × 10^6^ | 4.23 × 10^6^ | 6.80 × 10^6^ | | 4.40 × 10^6^ | | - | | - |
| lbp-2D | glcm | | DifferenceVariance | | 5.40 × 10^3^ | 3.45 × 10^2^ | - | | - | | - | | - |
| lbp-3D-k | glrlm | | LongRunHighGrayLevelEmphasis | | 2.16 × 10^1^ | 2.83 × 10^0^ | 2.13 × 10^1^ | | 2.88 × 10^0^ | | 2.14 × 10^1^ | | 2.95 × 10^0^ |
| lbp-3D-m1 | firstorder | | Kurtosis | | 4.16 × 10^0^ | 2.80 × 10^−1^ | - | | - | | - | | - |
|  | glcm | | ClusterShade | | - | - | - | | - | | 7.83 × 10^0^ | | 7.33 × 10^0^ |
| lbp-3D-m2 | firstorder | | Kurtosis | | 3.64 × 10^0^ | 3.24 × 10^−1^ | 3.66 × 10^0^ | | 3.14 × 10^-1^ | | 3.64 × 10^0^ | | 3.29 × 10^−1^ |
|  | glrlm | | ShortRunHighGrayLevelEmphasis | | - | - | 1.13 × 10^2^ | | 6.94 × 10^0^ | | - | | - |
| log-sigma-0-4492-mm-3D | firstorder | | Mean | | 1.75 × 10^0^ | 7.49 × 10^−1^ | - | | - | | 1.78 × 10^0^ | | 8.15 × 10^−1^ |
|  |  |  | RobustMeanAbsoluteDeviation | | - | - | - | | - | | - | | - |
| original | glrlm | | RunEntropy | | - | - | - | | - | | - | | - |
|  | shape | | LeastAxisLength | | 1.20 × 10^1^ | 2.51 × 10^0^ | 1.19 × 10^1^ | | 2.56 × 10^0^ | | 1.20 × 10^1^ | | 2.56 × 10^0^ |
|  |  |  | SurfaceArea | | 2.52 × 10^3^ | 8.57 × 10^2^ | 2.50 × 10^3^ | | 8.59 × 10^2^ | | 2.51 × 10^3^ | | 8.78 × 10^2^ |
|  |  |  | SurfaceVolumeRatio | | 1.08 × 10^0^ | 3.71 × 10^-1^ | 1.10 × 10^0^ | | 3.79 × 10^−1^ | | 1.09 × 10^0^ | | 3.92 × 10^−1^ |
| **Filter Type** | | **Feature Type** | | **Feature Name** | | **Fold 4** | | | | **Fold 5** | | | |
|  |  |  |  |  |  | **mean** | | **scale** | | **mean** | | **scale** | |
| exponential | | firstorder | | Energy | | - | | - | | 3.10 × 10^6^ | | 2.91 × 10^6^ | |
|  |  |  |  | Variance | | - | | - | | 2.72 × 10^1^ | | 2.40 × 10^1^ | |
|  |  | glcm | | SumEntropy | | 4.68 × 10^0^ | | 6.12 × 10^−1^ | | 4.66 × 10^0^ | | 6.00 × 10^−1^ | |
|  |  | glrlm | | RunVariance | | - | | - | | - | | - | |
| gradient | | firstorder | | Energy | | - | | - | | - | | - | |
| lbp-2D | | glcm | | DifferenceVariance | | - | | - | | - | | - | |
| lbp-3D-k | | glrlm | | LongRunHighGrayLevelEmphasis | | 2.15 × 10^1^ | | 2.95 × 10^0^ | | 2.14 × 10^1^ | | 2.83 × 10^0^ | |
| lbp-3D-m1 | | firstorder | | Kurtosis | | - | | - | | 4.15 × 10^0^ | | 2.65 × 10^−1^ | |
|  |  | glcm | | ClusterShade | | - | | - | | - | | - | |
| lbp-3D-m2 | | firstorder | | Kurtosis | | 3.64 × 10^0^ | | 3.32 × 10^−1^ | | - | | - | |
|  |  | glrlm | | ShortRunHighGrayLevelEmphasis | | - | | - | | - | | - | |
| log-sigma-0-4492-mm-3D | | firstorder | | Mean | | −1.79 × 10^0^ | | 8.16 × 10^−1^ | | −1.83 × 10^0^ | | 7.67 × 10^−1^ | |
|  |  |  |  | RobustMeanAbsoluteDeviation | | 1.72 × 10^0^ | | 4.00 × 10^−1^ | | - | | - | |
| original | | glrlm | | RunEntropy | | - | | - | | 6.03 × 10^0^ | | 2.60 × 10^−1^ | |
|  |  | shape | | LeastAxisLength | | - | | - | | - | | - | |
|  |  |  |  | SurfaceArea | | 2.52 × 10^3^ | | 8.57 × 10^2^ | | 2.50 × 10^3^ | | 8.59 × 10^2^ | |
|  |  |  |  | SurfaceVolumeRatio | | 1.08 × 10^0^ | | 3.71 × 10^−1^ | | 1.10 × 10^0^ | | 3.79 × 10^−1^ | |

**Datasheet S1 (available in the separate excel file).** Complete list of extracted radiomic features.

**References**

1. Van Griethuysen, J.J.; Fedorov, A.; Parmar, C.; Hosny, A.; Aucoin, N.; Narayan, V.; Beets-Tan, R.G.; Fillion-Robin, J.-C.; Pieper, S.; Aerts, H.J. Computational radiomics system to decode the radiographic phenotype. *Cancer Res.* **2017**, *77*, e104–e107.
2. Jenul, A.; Schrunner, S.; Liland, K.H.; Indahl, U.G.; Futsaether, C.M.; Tomic, O. RENT—Repeated Elastic Net Technique for Feature Selection. *IEEE Access* **2021**, *9*, 152333–152346, doi:10.1109/Access.2021.3126429.
3. Freund, Y.; Schapire, R.E. A decision-theoretic generalization of on-line learning and an application to boosting. *J. Comp. Sys. Sci.* **1997**, *55*, 119–139.
4. Pedregosa, F.; Varoquaux, G.; Gramfort, A.; Michel, V.; Thirion, B.; Grisel, O.; Blondel, M.; Prettenhofer, P.; Weiss, R.; Dubourg, V. Scikit-learn: Machine learning in Python. *J. Mach. Learn. Res.* **2011**, *12*, 2825–2830.
5. Nogueira, S.; Sechidis, K.; Brown, G. On the stability of feature selection algorithms. *J. Mach. Learn. Res.* **2017**, *18*, 6345–6398.
